# Transverse pion structure beyond leading twist in constituent models

**DOI:** 10.1140/epjc/s10052-016-4257-8

**Published:** 2016-07-22

**Authors:** C. Lorcé, B. Pasquini, P. Schweitzer

**Affiliations:** 1 Centre de Physique Théorique, École polytechnique, CNRS, Université Paris-Saclay, 91128 Palaiseau, France; 2 Dipartimento di Fisica, Università degli Studi di Pavia, Pavia, Italy; 3 Istituto Nazionale di Fisica Nucleare, Sezione di Pavia, Pavia, Italy; 4 Department of Physics, University of Connecticut, Storrs, CT 06269 USA; 5 Institute for Theoretical Physics, Tübingen University, Auf der Morgenstelle 14, 72076 Tübingen, Germany

## Abstract

The understanding of the pion structure as described in terms of transverse-momentum-dependent parton distribution functions (TMDs) is of importance for the interpretation of currently ongoing Drell–Yan experiments with pion beams. In this work we discuss the description of pion TMDs beyond leading twist in a pion model formulated in the light-front constituent framework. For comparison, we also review and derive new results for pion TMDs in the bag and spectator model.

## Introduction

The pion is one of the few hadrons, besides nucleon and nuclei, whose partonic structure can be studied, mainly thanks to the Drell–Yan process (DY) [1, 2] with pion beams impinging on nuclear targets [3–6]. DY data provide access to the twist-2 “collinear” parton distribution function (PDF) of the pion $f_1^a(x)$ [7–14] and more. In fact, the unpolarized DY cross section differential in the dilepton angular distribution, given in the Collins–Soper frame [15] by

1 $$\begin{aligned} \frac{\mathrm {d}\sigma }{\mathrm {d}\Omega } \propto \bigg ( 1 + \lambda \cos ^2 \theta + \mu \sin 2\theta \cos \phi + \frac{\nu }{2} \sin ^2 \theta \cos 2\phi \bigg ),\nonumber \\ \end{aligned}$$

provides also information on transverse-momentum-dependent parton distribution functions (TMDs). In the TMD factorization framework, the coefficient $\lambda$ is due to the twist-2 unpolarized TMD $f_1^q(x,p_T)$ and $1/Q^2$-suppressed terms, $\mu$ arises from certain twist-3 TMDs [16], $\nu$ is due to the naive time-reversal odd ($\mathsf T$-odd) Boer–Mulders function [17]. One important current development consists in extending the DY measurements to include polarization effects, which is being pursued with polarized proton beams at RHIC (BNL) [18] and pion beams impinging on polarized proton targets at COMPASS (CERN) [19, 20]. These experiments will test the TMD factorization approach, in particular the predicted sign change of naive $\mathsf T$-odd TMDs [21], and provide new insights on the nucleon structure.

In our context, the COMPASS program is of particular interest. It will give at the same time new insights on the pion structure at leading and subleading twist, and it will go far beyond what was learned from earlier Fermilab and CERN experiments [22–24] owing to the availability of a polarized target. Moreover, previous measurements suffered from limited statistics, and most of them found for instance a subleading-twist coefficient $\mu$ compatible with zero. Also with this respect new data from COMPASS may improve the situation [19].

Higher-twist PDFs and TMDs are of interest in their own right, as they provide a window on quark–gluon dynamics. By exploring the equations of motion (EOM) of QCD, higher-twist PDFs and TMDs can in general be decomposed into contributions from leading-twist, current quark mass terms and pure quark–gluon interaction-dependent (“tilde”) terms. An interesting question is how such genuine QCD interaction-dependent terms are modeled in constituent frameworks, which for our purposes are defined as models without explicit gluon degrees of freedom.

In a previous study we addressed this question in the context of unpolarized nucleon PDFs and TMDs [25]. We have shown that internally consistent descriptions of the unpolarized leading- and higher-twist PDFs and TMDs are possible using several constituent model approaches. The respective effective interactions mimic in various ways the QCD quark–gluon interactions, giving rise to non-trivial tilde terms in some models. To which extent constituent models can provide phenomenologically reliable estimates for higher-twist effects remains to be tested. At least an encouraging agreement was observed [25] in the case of the nucleon twist-3 PDF $e^q(x)$ of which recently a first extraction became available [26].

In this work we will present a study for the pion case. The main scope is to prepare an understanding of $\mathsf T$-even pion TMDs at leading and especially subleading twist in the framework of constituent models which can be tested and used in future phenomenological applications to analyze and interpret first data. Our particular focus will be on critically reviewing the internal consistency of the models, and assess their range of applicability. We will also investigate how the genuine higher-twist terms are modeled in different effective-model frameworks. Our focus will be on the aspects peculiar to the meson sector, i.e. on aspects related to the modeling of 2-body dynamics of the $q\bar{q}$-pair in the pion as opposed to the modeling of 3-body dynamics in the nucleon state investigated in prior work [25].

The three models discussed in this work are the light-front constituent model (LFCM), the bag, and the spectator model. All results for higher-twist TMDs are new and original in the LFCM and bag model. In the spectator model analytical expressions for twist-3 pion TMDs were quoted in the literature, but to the best of our knowledge they were neither evaluated nor were their properties discussed. We discuss and compare the results from the different models with the goal to establish differences and common features of constituent frameworks of the pion structure.

It is important to keep in mind that none of these models accounts for the perhaps most important feature of the pion, namely its nature as Goldstone boson associated with spontaneous chiral symmetry breaking. Instead, the models discussed in this work treat the pion on the same footing as all other hadrons, i.e. as a particle composed of the respective constituent degrees of freedom. In our assessment of the applicability of the models, we shall also discuss the rational for this approach. A study of twist-2 pion TMDs in a chiral (Nambu-Jona-Lasinio) model was presented in Ref. [27].

The outline is as follows. In Sect. 2 we define and discuss the properties of pion TMDs in constituent models. In Sect. 3 we study pion TMDs in the LFCM. In Sect. 4 we review the descriptions of pion TMDs in the bag and spectator model. In Sect. 5 we present the numerical results from the different models and compare them to nucleon TMDs. Finally, Sect. 6 contains the conclusions. Technical details are collected in the appendices.

## $\mathsf T$-even pion TMDs in quark models

TMDs are described in terms of quark correlators. In constituent approaches without explicit gluon degrees of freedom, the Wilson lines of QCD reduce to unit matrices in color space. As a result $\mathsf T$-odd TMDs are absent, and only $\mathsf T$-even TMDs appear. The structure of a spin-zero hadron, like the pion, is described in terms of four TMDs,

2a $$\begin{aligned}&\int \frac{\mathrm {d}z^-\mathrm {d}^2z_T}{2(2\pi )^3} \, e^{i p \cdot z} \, \langle P|\overline{\psi }(0)\gamma ^+ \psi (z)|P\rangle |_{z^+=0} = f_1^q(x,p_T), \end{aligned}$$

2b $$\begin{aligned}&\int \frac{\mathrm {d}z^-\mathrm {d}^2z_T}{2(2\pi )^3} e^{i p \cdot z} \langle P | \overline{\psi }(0)\;\mathbbm {1}\; \psi (z) | P\rangle |_{z^+=0} \nonumber \\&\quad = \frac{m_\pi }{P^+}\,e^q(x,p_T), \end{aligned}$$

2c $$\begin{aligned}&\int \frac{\mathrm {d}z^-\mathrm {d}^2z_T}{2(2\pi )^3} e^{i p \cdot z} \langle P | \overline{\psi }(0)\gamma _T^j \psi (z) | P\rangle |_{z^+=0} \nonumber \\&\quad = \frac{p_T^j}{P^+}\,f^{\perp q}(x,p_T), \end{aligned}$$

2d $$\begin{aligned}&\int \frac{\mathrm {d}z^-\mathrm {d}^2z_T}{2(2\pi )^3} e^{i p \cdot z} \langle P | \overline{\psi }(0)\gamma ^- \psi (z) | P\rangle |_{z^+=0} \nonumber \\&\quad = \frac{m_\pi ^2}{(P^+)^2}f_4^q(x,p_T). \end{aligned}$$

Here $|P\rangle$ is a pion state with 4-momentum *P*, *q* is a flavor index for the quark and antiquark contribution and $m_\pi$ is the pion mass. We use light-front coordinates $a^\pm = (a^0 \pm a^3)/\sqrt{2}$, ${\varvec{a}}_T=(a^1,a^2)$ with $a_T\equiv |{\varvec{a}}_T|$ and the metric is $a\cdot b$ = $a^+b^-+a^-b^+-{\varvec{a}}_T\cdot {\varvec{b}}_T$ with $\mathrm {d}^4 z = \mathrm {d}z^+\mathrm {d}z^-\mathrm {d}^2 z_T$. The model results generically refer to a low (“hadronic”) normalization scale below 1 GeV [28–30]. Integrating Eq. (2) over $\varvec{p}_T$ provides the definition of the corresponding PDFs. Note in particular that because of the explicit $p^j_T$ factor in Eq. (2c) there does not exist any PDF counterpart to $f^{\perp q}(x,p_T)$. One can, however, formally define $f^{\perp q}(x)\equiv \int \mathrm {d}^2p_T\,f^{\perp q}(x,p_T)$.

Sum rules are of particular importance when testing the consistency of models. Let $N_q$ be the valence number of flavor *q*, which is for instance $N_u=N_{\bar{d}}=1$ in $\pi ^+$. The sum rules are given by

3a $$\begin{aligned}&\int \mathrm {d}x\,f_1^q(x) = N_q\,, \end{aligned}$$

3b $$\begin{aligned}&\sum _q\int \mathrm {d}x\;x\,f_1^q(x) = 1 \,, \end{aligned}$$

3c $$\begin{aligned}&\sum _q\int \mathrm {d}x\,e^q(x) = \frac{\sigma _{\pi }}{m_q}\,, \end{aligned}$$

3d $$\begin{aligned}&\int \mathrm {d}x\;x\;e^q(x) = \frac{m_q}{m_\pi }\;N_q\,, \end{aligned}$$

3e $$\begin{aligned}&2\int \mathrm {d}x\,f_4^q(x) = N_q. \end{aligned}$$

The valence number sum rule (3a) is the same in QCD and constituent models, but the momentum sum rule (3b) is saturated solely by valence degrees of freedom in constituent models at the initial scale (with the exception of the spectator model which we will discuss in detail). Equation (3c) formally relates $e^q(x)$ to the sigma term [31, 32], which corresponds to the scalar form factor $\sigma (t)$ at zero-momentum transfer. The sigma term of the pion is given by $\sigma _\pi = \frac{1}{2}\,m_\pi$ in the leading order of the chiral expansion. Since $m_\pi ^2\propto m_q$ owing to the Gell-Mann–Oakes–Renner relation, the sum rule (3c) for the pion diverges like $1/m_\pi$ in the chiral limit. The Jaffe–Ji sum rule (3d) connects the first moment of $e^q(x)$ to the current quark mass $m_q$ in QCD, or the constituent (or effective) mass in models [25, 33]. In the chiral limit this sum rule goes to zero like $m_\pi$. The sum rule (3e) formally arises from the normalization of the minus-component of the vector current, just as (3a) arises from the normalization of its plus-component. The validity of (3e) is subtle, both in QCD and in quark models [25], as we shall discuss in Sects. 3 and 4. In Eq. (3c) and throughout this work, we neglect isospin-violating effects and assume $m_q=m_u=m_d$ for current or constituent quark masses. Unless otherwise stated, we will refer to the distributions in positive pions using the notation $j^{u}_{\pi ^+}(x,p_T)\equiv j^q(x,p_T)$, where $j_{\pi ^{+}}^{u}=j_{\pi ^{+}}^{\bar{d}} =j_{\pi ^{-}}^{d}=j_{\pi ^{-}}^{\bar{u}} =2 \,j_{\pi ^{0}}^{u} = 2 \,j_{\pi ^{0}}^{\bar{u}} =2 \,j_{\pi ^{0}}^{d} = 2 \,j_{\pi ^{0}}^{\bar{d}}$ holds due to isospin symmetry and charge conjugation, and $j^q(x,p_T)$ denotes a generic TMD.

Positivity inequalities provide another important test, although they can be spoiled in QCD already at leading twist (let alone at twist-4) due to subtractions in the renormalization procedure. In consistent models one expects [25]

4a $$\begin{aligned} f_1^q(x,p_T) \ge 0, \end{aligned}$$

4b $$\begin{aligned} f_4^q(x,p_T) \ge 0. \end{aligned}$$

In approaches without explicit gauge-degrees of freedom, the quark correlator of a spin-zero (or unpolarized) hadron has a general Lorentz decomposition in terms of three independent amplitudes parametrized in terms of four TMDs. In such situations “quark-model Lorentz-invariance relations (qLIRs)” arise [34].^1^ In our case, the qLIR is given by [25]

5 $$\begin{aligned} f_4^q(x) = \frac{1}{2}\,f_1^q(x)+\frac{\mathrm {d}}{\mathrm {d}x}f^{\perp q(1)}(x), \end{aligned}$$

with $$f^{\perp q(1)}(x)=\int \mathrm {d}^2p_T\,\tfrac{p^2_T}{2m_\pi ^2}f^{\perp q}(x,p_T)$$.

It is important to remark that $f_1^q$ and the twist-3 pion TMDs $e^q$ and $f^{\perp q}$ can be accessed in DY [16], but not the twist-4 TMD $f^q_4$, which therefore has to be considered as an academic object. Nevertheless $f^q_4$ completes the description of the quark correlator through twist-4 [37], and Eq. (5) is of value as it provides a powerful test for the theoretical consistency of a model.

Next, let us state the relations which result from employing the EOMs

6a $$\begin{aligned} x\,e^q(x,p_T)&= x\,\tilde{e}^q(x,p_T) + \frac{m_q}{m_\pi }\,f_1^q(x,p_T),\end{aligned}$$

6b $$\begin{aligned} x\,f^{\perp q}(x,p_T)&= x\,\tilde{f}^{\perp q}(x,p_T) + f_1^q(x,p_T),\end{aligned}$$

6c $$\begin{aligned} x^2f^q_4(x,p_T)&= x^2\tilde{f}_4^q(x,p_T) + \frac{p_T^2+m_q^2}{2m_\pi ^{2}}\;f_1^q(x,p_T). \end{aligned}$$

In QCD the tilde terms are expressed in terms of quark–gluon–quark correlators. In quark models, they still denote “interaction-dependent terms” which arise from applying the respective model EOMs.

## Pion structure in the LFCM

In this section we discuss pion TMDs in the LFCM. We first derive the general expressions for the subleading-twist TMDs in leading order of the Fock-space expansion for the pion, and discuss the consistency of the approach. We then introduce the phenomenological model for the light-front wave-functions (LFWFs) which we will employ later to obtain definite predictions.

### General formalism

The formalism for the calculation of the unpolarized higher-twist $\mathsf T$-even TMDs in the light-front framework has been discussed in Ref. [25], with an explicit application to the nucleon. The same approach is adopted here in the case of pion. We recall that in light-front quantization the Fock-space expansion of the hadron states is performed in terms of free on-mass-shell parton states with the essential QCD bound-state information encoded in the LFWF. The $q\bar{q}$ component of the light-front state of the pion can be written as

7 $$\begin{aligned} {\vert \pi (\tilde{P})\rangle }_{q\bar{q}} = \sum _{\lambda _1,\lambda _2} \int \mathrm {d}[1]\,\mathrm {d}[2]\, \Psi ^{q \bar{q}}_{\lambda _1\lambda _2}(r_1,r_2) |\lambda _i, \tilde{p}_i \rangle \, , \end{aligned}$$

where $\Psi ^{q \, \bar{q}}_{\lambda _1\lambda _2}$ is the $q\bar{q}$-LFWF with $\lambda _1$ ($\lambda _2$) and *q* ($\bar{q}$) referring to the light-front helicity and flavor of quark (antiquark), respectively. The LFWF includes an isospin factor $T_\pi$ which projects onto the different members of the isotriplet of the pion, i.e. $T_\pi =\sum _{\tau _1,\tau _{2}}\langle \tfrac{1}{2} \tau _1 \tfrac{1}{2} \tau _{2}| 1 \tau _\pi \rangle$ with $\tau _1, \tau _{2}$ and $\tau _\pi$ the isospin of the quark, antiquark, and pion state, respectively. In Eq. (7) $r_i=(x_i M_{0} , {\varvec{p}}_{Ti})$, and $M_0$ denotes the mass of the non-interacting $q\bar{q}$ state. Furthermore, we introduced the notation $\tilde{p}=(p^+,{\varvec{p}}_T)$ for a generic light-front momentum variable *p*. Since momentum conservation implies ${\varvec{p}}_{T1}+{\varvec{p}}_{T2}=\varvec{0}_T$ and $x_1+x_2=1$, the LFWF actually depends only on the variables $\bar{x}=x_1$ and ${\varvec{\kappa }}_{T}={\varvec{p}}_{T1}$. The integration measure in Eq. (7) is defined as

8 $$\begin{aligned} \mathrm {d}[1]\,\mathrm {d}[2]= & {} \frac{\mathrm {d}x_1\,\mathrm {d}x_2 }{\sqrt{x_1x_2}}\,\delta \!\left( 1-x_1-x_2\right) \nonumber \\&\times \frac{\mathrm {d}^2 p_{T1}\,\mathrm {d}^2p_{T2}}{2(2\pi )^3} \, \delta ^{(2)}\!\left( {\varvec{p}}_{T1}+{\varvec{p}}_{T2}\right) , \end{aligned}$$

so that we can write

9 $$\begin{aligned}&\int \mathrm {d}[1]\,\mathrm {d}[2]\,F(x_1,{\varvec{p}}_{T1},x_2,{\varvec{p}}_{T2}) \nonumber \\&\quad =\int \frac{\mathrm {d}\bar{x}}{\sqrt{\bar{x}(1-\bar{x})}} \,\frac{\mathrm {d}^2\kappa _{T}}{2(2\pi )^3}\, F( \bar{x},{\varvec{\kappa }}_T, 1-\bar{x}, -{\varvec{\kappa }}_T). \end{aligned}$$

The pion TMDs are given by the expressions

10a $$\begin{aligned}&f_1^q(x,p_T)= \mathcal{P}^q(\tilde{p}),\end{aligned}$$

10b $$\begin{aligned}&x\,e^q(x,p_T)=\frac{m_q}{m_\pi }\, \mathcal{P}^q(\tilde{p}),\end{aligned}$$

10c $$\begin{aligned}&x\,f^{\perp q}(x,p_T)=\mathcal{P}^q(\tilde{p}),\end{aligned}$$

10d $$\begin{aligned}&x^2\,f^q_{4}(x,p_T)=\frac{p_T^2+m_{q}^2}{2m_\pi ^2}\,\mathcal{P}^q(\tilde{p}), \end{aligned}$$

which formally coincide with the expressions for the unpolarized nucleon TMDs [25], except that the quark density operator $\mathcal{P}^q(\tilde{p})$ is evaluated in the pion states, which are given in terms of the pion LFWFs by

11 $$\begin{aligned} \mathcal{P}^q(\tilde{p})= & {} \sum _{\lambda _1,\lambda _2} | \Psi ^{q \bar{q}}_{\lambda _1\lambda _{2}}( \tilde{p} ) |^2. \end{aligned}$$

Equations (10a)–(10d) are model-independent in the sense that they are valid in every light-front approach in which the Fock-space expansion includes the leading (“valence”) sector and truncates higher Fock-space components.

### Internal consistency of the approach

Let us now test the internal consistency of the approach. From Eqs. (10a)–(10d) we obtain the relations

12a $$\begin{aligned}&x\,e^q(x,p_T)=\frac{m_q}{m_\pi }\,f_{1}^q(x,p_T),\end{aligned}$$

12b $$\begin{aligned}&x\, f^{\perp q}(x,p_T)=f_{1}^q(x,p_T),\end{aligned}$$

12c $$\begin{aligned}&x^2\,f_4(x,p_T) = \frac{p_T^2+m_q^2}{2m_\pi ^{2}}\,f_1^q(x,p_T), \end{aligned}$$

which coincide with the EOM relations (6a)–(6c), respectively, with vanishing tilde terms as expected for free on-shell partons described in terms of LFWFs.

The valence number sum rule (3a) and the momentum sum rule (3b) are satisfied in the LFCM by construction. As a consequence of Eq. (12b), one finds $\int \mathrm{d}x \, xf^{\perp q}(x)=N_q$ and $\sum _q\int \mathrm{d}x \, x^2 f^{\perp q}(x)=1$.

The sum rules for the first and second Mellin moment of $e^q(x)$ in Eqs. (3c) and (3d) are valid with the proofs analog to the nucleon case [25]. The sum rule (3d) also follows directly from Eq. (12a), which in addition implies a sum rule for the second moment $\sum _q\int \mathrm{d}x \, x^2 e^q(x)=m_q/m_\pi$.

The sum rule (3e) for $f_4(x)$ is not supported in the LFCM of the pion, and also the qLIR (5) is not valid. These observations were also made in the nucleon case [25] and are related to each other. The fact that the same features occur in the pion (2-body) and nucleon (3-body) case, indicates that this is not an artifact but a general property of LFCMs. To ensure the compliance with the sum rule (3e) it is necessary to consider zero modes in the light-front quantization [38] or to include higher light-front Fock states [39]. These considerations are beyond the scope of LFCMs based on the minimal Fock space, so that both the sum rule (3e) and the qLIR (5) are consequently not satisfied [25]. The LFCM of the pion complies, however, with positivity (4a), (4b).

Thus, the LFCM is internally consistent. It satisfies all general relations except for the sum rule (3e) and the qLIR (5) which are beyond the scope of this approach, and both related to the academic twist-4 PDF $f_4^q(x)$ such that it has no relevance for practical applications.

### Phenomenological model for LFWF

To obtain definite predictions one has to choose a specific model for LFWFs. In this work we choose the pion LFWFs proposed in Refs. [40, 41]. One could include the effects of confinement in the light-cone approach [42], but the phenomenological LFWFs of [40, 41] provide already a phenomenologically acceptable description. They were applied in Refs. [30, 43] to the calculations of leading-twist $\mathsf T$-even and $\mathsf T$-odd TMDs, and generalized parton distributions of the pion. For completeness, we briefly review this model.

The explicit expression for the momentum-dependent part of the LFWF reads

13 $$\begin{aligned} {\tilde{\psi }}_\pi (\bar{x},{\varvec{\kappa }}_{T }) = \sqrt{2(2\pi )^3}\;\sqrt{ \frac{M_0(\bar{x},{\varvec{\kappa }}_T)}{4~\bar{x} (1-\bar{x})}}\; \frac{e^{-{\varvec{\kappa }}^2/2\beta ^2}}{\pi ^{3/4}\beta ^{3/2}}\,, \end{aligned}$$

where ${\varvec{\kappa }}=(\varvec{\kappa }_T, \kappa _z)$ is the quark three-momentum, with

14 $$\begin{aligned} \kappa _{z}=M_0(\bar{x},{\varvec{\kappa }}_{T})\,(\bar{x}-\tfrac{1}{2}), \end{aligned}$$

and the free invariant mass squared is given by

15 $$\begin{aligned} M_0^2(\bar{x},{\varvec{\kappa }}_{T})= \frac{m^2_q+\kappa ^2_{T}}{\bar{x}(1-\bar{x})}. \end{aligned}$$

The LFWF (13) depends on the free parameter $\beta$ and the quark mass $m_q$, which have been fitted to the pion charge radius and decay constant. In particular, we take $m_q=0.250$ GeV and $\beta =0.3194$ [40]. For the spin-dependent part of the LFWF we refer to the derivation in Ref. [30].

The results obtained with this pion LFWF model will be discussed, and confronted with other models in Sect. 5.

## Pion structure in bag and spectator model

In this section we discuss pion TMDs in two other models, the bag and spectator model. We focus on physical aspects and internal consistency in these approaches, and skip technical details which are collected in Appendix A and Appendix B.

### Bag model framework

The bag model describes hadrons in terms of *n* free quark and/or antiquark constituents confined inside a spherical cavity of radius $R_\mathrm{bag}$ by appropriate boundary conditions [44]. In its simplest version $\pi$- and $\rho$-mesons are mass-degenerate, as it makes no difference whether a $\bar{q} q$-pair is placed in an *s*-wave with aligned or anti-aligned spins. This unrealistic situation can be improved [45] by invoking a gluon-exchange potential (which is an intrinsic property of the bag wave-function, and different from the gluonic effects related to initial- or final-state interactions [46] that give rise to $\mathsf T$-odd TMDs). Also “center-of-mass corrections” were used to construct wave-packet superpositions of static bag solutions with naturally light pion masses [47] that met phenomenological success [48]. A bag model version constructed to comply with chiral symmetry is the “cloudy bag” [49].

In this work we use the simple MIT bag model with massless quarks. At first glance this seems not to fit in the generic picture of massive, effective, constituent degrees of freedom. But if desired, one can introduce a quark mass parameter with numerical but no conceptual differences in the model, with a value around $m_q \sim 120\,\mathrm MeV$ [50] which is natural from the point of view of the constituent picture (although also smaller values were discussed in the literature). More importantly, the quantum numbers of hadrons are determined by a fixed number of valence (quark, antiquark) degrees of freedom, which allows one to classify the bag model as a constituent framework. This approach is therefore sufficient for our purposes to investigate generic features of TMDs in constituent models. The bag model expressions for $f_1^q(x,p_T)$, $e^q(x,p_T)$, $f^{\perp q}(x,p_T)$, and $f_4^q(x,p_T)$ in the pion are given in Appendix A.1.

Keeping in mind the well-known general shortcomings, the description has to be considered as consistent: the bag model TMDs satisfy the sum rules^2^ (3a), (3b), (3e). The sum rules (3c), (3d) are more subtle, and discussed in Appendix A.2 where we show that they are consistently satisfied in the model albeit in a quite different manner compared to QCD. The bag results satisfy the inequalities (4a), (4b). As a last and stringent consistency check of the description of higher-twist TMDs, we remark that the bag model satisfies the qLIR (5). This was proven analytically for nucleon TMDs in [25]. The proof can be carried over to the pion case such that also pion TMDs comply with Eq. (5). The EOM relations (6a)–(6c) hold with non-zero interaction-dependent tilde terms which are due to bag boundary effects [25, 31].

Overall we find that the bag model description of higher-twist TMDs is internally consistent within the model, although not all features of the model are consistent with QCD. The PDFs in the bag model exhibit also interesting symmetry properties which we discuss in detail in Appendix A.3. We shall return to the bag model and discuss further properties of TMDs and numerical results in Sect. 5.

### Spectator model

In the spectator approach the pion structure is modeled in terms of an effective pion–quark–spectator vertex. The spectator has the quantum numbers of an antiquark but, constituting an effective degree of freedom, it could in principle have a different mass. We distinguish the spectator mass $M_R$ and constituent mass $m_q$ in the formulas in Appendix B, but we set them equal in the final results. This choice is closest to the spirit of constituent models where, after the active quark is struck, one would identify the “remainder” with an antiquark. This is of course not a necessary step. However, the rational for working with a distinct effective degree of freedom is less convincing than in the nucleon case, where the “remainder” has the quantum numbers of diquarks, i.e. effective bosonic degrees of freedom whose masses are a priori free parameters which cannot be associated with the constituent quark mass. This approach was used to compute the pion TMDs $f_1^q(x,p_T)$, $f^{\perp q}(x,p_T)$, $e^q(x,p_T)$ in Ref. [51]. In Appendix B.1 we review the expressions for these TMDs, and derive also the spectator model expression for $f_4^q(x,p_T)$.

**Fig. 1:**
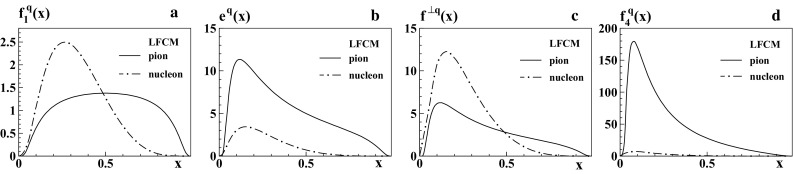
LFCM results for PDFs as functions of *x*. The *solid curves* correspond to the pion results with the LFWF of Ref. [30] for the *u*-flavor in $\pi ^+$. The *dashed-dotted curves* show for comparison the corresponding results for the *d*-flavor PDFs in the proton in the LFCM of Ref. [25], which have the same normalization for $f_1^q(x)$

Let us now concentrate on discussing the consistency of the approach which, regarding the sum rules (3a)–(3e), is conceptually the same in the spectator model of the pion as in the spectator model of the nucleon [25]. The valence sum rule (3a) is satisfied in this model by construction, as the normalization of the effective vertex is chosen adequately. In contrast, the momentum sum rule (3b) is not valid for any choice of model parameters: one obtains less than unity in Eq. (3b). In a specific parametric limit, one obtains a quasi-model-independent result that the valence quark and antiquark carry $\frac{2}{3}$ of the pion’s momentum. Such “$\frac{2}{3}$-paradoxes” have a long history in the literature and illustrate that the model is incomplete; see the detailed discussion in Appendix B.2.

The sum rules (3c) and (3d) for $e^q(x)$ do not hold in the spectator model of the pion. This is apparent from the fact that the first and second moments in Eqs. (3c) and (3d) should be positive, while $e^q(x)$ is negative in this model as discussed in Appendix B.3.

Also the sum rule (3e) for $f^q_4(x)$ is not satisfied in the spectator model, but this has a different origin. Both sum rules (3a) and (3e) can be traced back to the conservation of the Noether vector current. The form factors, which are introduced in an ad hoc manner to describe the effective vertex (see Eq. (39) in Appendix B.1) in general violate current conservation. It is therefore possible to satisfy (3a) or (3e) but not both sum rules simultaneously.

The spectator model complies with the positivity requirement (4a) for $f_1^q(x)$, and satisfies the inequality (4b) for $f_4^q(x)$ provided one choses the model parameters appropriately; see Appendix B.3. As a last test of the spectator model, we notice that the qLIR (5) is satisfied. The proof for that can be carried over from the nucleon case [25].

Finally, let us remark that the EOM relations (6a)–(6c) hold in the spectator model of the pion with the tilde terms arising due to the off-shellness of the quark, analog to the nucleon case [25]. Remarkably, in the pion the off-shellness effects and hence the tilde terms are large when one identifies the mass of the spectator particle with the constituent quark mass. This is discussed in detail in Appendix B.4.

## Numerical results

In order to discuss the model results, we first focus on the integrated TMDs in the three models in Sects. 5.1–5.3. Then we discuss the $p_T$-dependence of the TMDs in Sect. 5.4.

**Fig. 2:**
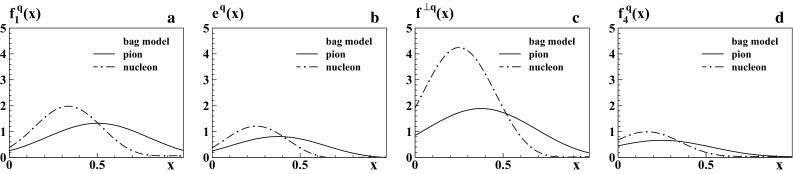
Bag model results for pion PDFs (*solid lines*) as functions of *x* at low scale: **a** $f_1^q(x)$, **b** $e^q(x)$, **c** $f^{\perp q}(x)$, **d** $f_4^q(x)$. The pion results (*solid curves*) refer e.g. to the *u*-flavor in $\pi ^+$. For comparison the corresponding nucleon PDFs from Ref. [25] are shown (*dashed-dotted curves*) for *d*-flavor in the proton, such that in panel **a** both curves are normalized to unity (cf. footnote 3)

### Integrated TMDs in LFCM

In Fig. 1 we show the LFCM results for the integrated TMDs $f_1^q(x)$, $e^q(x)$, $f^{\perp q}(x)$, and $f_4^q(x)$ of the pion in comparison with the corresponding results for the down quark in the nucleon, obtained from the three-quark LFWF of Refs. [25, 52]. In the LFCM the distribution of quark with longitudinal momentum fraction *x* is equal to the distribution of the corresponding antiquark with longitudinal momentum fraction $1-x$, i.e. for instance in $\pi ^+$ we have $f_{1}^{u}(x)=f_{1}^{\bar{d}}(1-x)$. Furthermore, one has the relation $f_{1}^{\bar{d}}(x)=f_{1}^{u}(x)$ which gives as final result a momentum distribution symmetric with respect to $x=\frac{1}{2}$. The shape of the unpolarized momentum distributions for the pion and proton is quite different, reflecting the different valence-quark structure of the hadrons. For the proton, the unpolarized momentum distribution of the valence quark is peaked at $x \approx 1/3$, while for the pion it reaches its maximum at $x=1/2$.

The twist-3 distributions of both the pion and the nucleon can be expressed in terms of the unpolarized momentum distribution as in Eqs. (10a)–(10d), with the corresponding hadron mass and constituent quark mass.^3^ The small value of the pion mass accounts for the enhancement of the $e^q$ and $f_4^q$ parton distributions with respect to $f^q_1$, which is much more pronounced than in the case of the nucleon, especially for $f_4^q$.

Finally, let us remark that in the LFCM it is possible to evaluate also inverse moments. For instance, the inverse moment

16 $$\begin{aligned} \langle x^{-1}\rangle _q = \int _0^1\mathrm {d}x\;\frac{f_1^q(x)}{x} \end{aligned}$$

exists and is well defined in the LFCM. In fact, thanks to the EOM relations (6a) and (6b) it is related to the first moment of $f^{\perp q}(x)$ or the first moment of $e^q(x)$ (and by means of (3c) also to $\sigma _\pi$) in this model. Such inverse moments have been discussed in the literature [53] in the context of a modern reformulation of the Weisberger sum rule [54]. In general, in QCD as well as in the other models considered in this work, such inverse moments diverge and are ill-defined, so it is noteworthy that the LFCM provides a framework where they can be evaluated—giving the opportunity to study sum rules based on inverse moments. We will not pursue this line further in this work, and we only remark that numerically one obtains

17 $$\begin{aligned} \langle x^{-1}\rangle _q = N_q\times {\left\{ \begin{array}{ll} 2.82\, &{}\quad \text{ for } \text{ the } \text{ pion } \text{(this } \text{ work), } \\ 3.97\, &{}\quad \text{ for } \text{ the } \text{ nucleon, } \text{ Ref. } \text{[25]. } \end{array}\right. } \end{aligned}$$

**Fig. 3:**
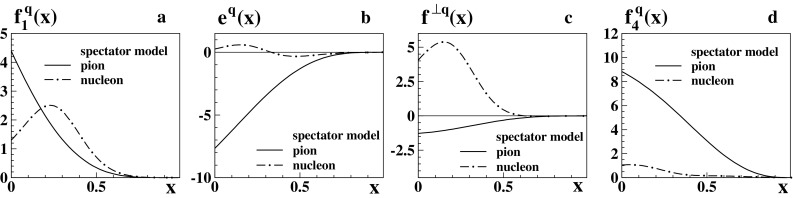
Results for pion PDFs (*solid lines*) from the spectator model as functions of *x* at low scale: **a** $f_1^q(x)$, **b** $e^q(x)$, **c** $f^{\perp q}(x)$, **d** $f_4^q(x)$. The pion results refer e.g. to the *u*-flavor in $\pi ^+$. For comparison the corresponding nucleon integrated TMDs from Ref. [25] are shown (*dashed-dotted curves*) for *d*-flavor in the proton, such that in panel **a** both curves are normalized to unity

### Integrated TMDs in bag model

The numerical results for the integrated pion TMDs from the bag model are shown in Fig. 2 in comparison to the results from the nucleon in this model [25, 55]. For $f_1^q(x)$ the results are qualitatively similar in shape and magnitude to those from the LFCM. But for $e^q(x)$, $f^{\perp q}(x)$, and $f_4^q(x)$ the bag model predicts much smaller distributions than the LFCM. This can be understood by means of the sum rules. In fact, $f_1^q(x)$ obeys the sum rules (3a) and (3b) which dictate comparable magnitudes in all quark models. On the other hand, the Jaffe–Ji sum rule (3d) does not place the same constraints regarding the magnitude of $e^q(x)$ in all models. The second moment of $e^q(x)$ is sizable in the LFCM because the constituent mass $m_q=250$ MeV enters the normalization of this sum rule in the LFCM. In contrast to this, the quarks in the bag model are massless and the sum rule (3d) is realized differently, see Appendix A.2, due to the different EOMs in the bag model. Another principal difference is that the TMDs of the pion and nucleon have the same order of magnitude in the bag model in contrast to the LFCM.

There are several interesting observations, which we summarize here leaving the details to Appendix A.3. In the bag model $f_1^q(x)$ exhibits a global maximum at $x_\mathrm{max}\approx \frac{1}{n}$ where *n* is the number of constituents, and shows an approximate reflection symmetry $f_1^q(x) \approx f_1^q(2x_\mathrm{max}-x)$, which is satisfied numerically (for the pion with $n=2$) with an accuracy better than $\mathcal{O}(1\,\%)$ in the valence-*x* region. As a consequence of this symmetry the unpolarized distribution in the pion is smaller and broader than that in the nucleon, where $f_1^q(x)$ is approximately symmetric with respect to its peak at $x_\mathrm{max}\approx \frac{1}{3}$. These are natural features in a system made of *n* constituents each one carrying on average about $x\sim \frac{1}{n}$ of the hadron momentum. With increasing *n* one would expect the distributions to exhibit narrower peaks around their maxima, as we observe. We remark that $f_4^q(x)$ has similar properties to $f_1^q(x)$, except that this PDF peaks at a different value $x_\mathrm{max}\approx \frac{1}{2n}$ and exhibits an approximate symmetry around this value; see Appendix A.3.

For pions the approximate symmetry $f_1^q(x) \approx f_1^q(1-x)$ implies that $f_1^q(x)$ has as much support at unphysical $x>1$ as in the region $x<0$ where it would describe minus the distribution of antiquarks according to $f_1^{\bar{q}}(x)=-f_1^q(-x)$. If we are willing to accept the spurious contributions at $x>1$ as a bag artifact (which can be remedied by adequate projection techniques), then we recognize that the pion has no sea quarks in the bag model, besides a spurious bag artifact contribution. This is a qualitatively and quantitatively different situation than in the nucleon, where $f_1^q(x)$ peaks around $x_\mathrm{max}\approx \frac{1}{3}$ and the bag generates, through the symmetry $f_1^q(x) \approx f_1^q(\frac{2}{3}-x)$, sizable sea quark contributions in the nucleon which violate positivity (4a).

With this last observation one arrives (somewhat paradoxically in view of the reservations regarding chiral symmetry) at the conclusion that the bag seems “better suited” for the description of the pion structure than the nucleon structure, as the problem of unphysical sea quarks does not appear in the pion case.

### Integrated TMDs in spectator model

In Fig. 3 we compare the integrated TMDs $f_1^q(x)$, $e^q(x)$, $f^{\perp q}(x)$, and $f_4^q(x)$ from the pion spectator model with the parameter fixing as described in Appendix B.3 to the results in the nucleon case obtained in [25, 51]. Interestingly, and in contrast to other models and to the nucleon case in the spectator model, the integrated pion TMDs do not exhibit a global extremum at finite *x*, but at the boundary value $x=0$. The predictions for the functions $e^q(x)$ and $f^{\perp q}(x)$ of the pion and nucleon differ significantly in this model. Although the description of these TMDs is conceptually the same (one basically deals with the same effective diagram in the “crossed channel” [51]), this is a consequence of the different parameters and the different relative size of off-shellness effects in pion and nucleon; see Appendix B.4.

### ${\varvec{p}}_T$-dependence in models

In this section we turn our attention to the $p_T$-dependence of the TMDs. Let us define the mean transverse momenta $(n=1)$ and the mean squared transverse momenta $(n=2)$ in a generic TMD $j(x,p_T)$ as follows:

18 $$\begin{aligned} \langle p_{T}^n\rangle = \frac{\int \mathrm {d}x\int \mathrm {d}^2p_T \,p_T^n\,j(x,p_T)}{\int \mathrm {d}x\int \mathrm {d}^2p_T \,j(x,p_T)}. \end{aligned}$$

If a TMD had exactly Gaussian $p_T$-dependence one would find for the ratio

19 $$\begin{aligned} R_G = \frac{2}{\sqrt{\pi }}\;\frac{\langle p_T\rangle }{\langle p_T^2\rangle ^{1/2}} \end{aligned}$$

the result $R_G=1$. This has been occasionally used as a quick test to see to which extent a model supports Gaussian $p_T$-behavior [28] which is observed phenomenologically in many DIS reactions [56, 57]. However, one should use such tests with caution as the following results from the LFCM show.

**Table 1:** (a) $\langle p_T\rangle$ and $\langle p_T^2\rangle ^{1/2}$ in units of GeV as defined in Eq. (18), and the ratio $R_G$ as defined in (19) for pion TMDs from LFCM. (b) The Gaussian widths $\langle p_{T,v}^2\rangle ^{1/2}$ defined in (20) in GeV for pion TMDs at $x_v=0.5$ from LFCM, spectator and bag model

(a) Pion	LFCM		
TMD	$\langle p_T\rangle$	$\langle p_T^2\rangle ^{1/2}$	$R_G$
$f_{1}^{q}$	0.28	0.32	0.99
$e^{q}$	0.26	0.30	0.99
$f^{\perp q}$	0.26	0.30	0.99
$f_{4}^{ q}$	0.30	0.33	0.98

(b) Pion	LFCM	Bag	Spectator
TMD	$\langle p_{T,v}^2\rangle ^{1/2}$	$\langle p_{T,v}^2\rangle ^{1/2}$	$\langle p_{T,v}^2\rangle ^{1/2}$
$f_1^{q}$	0.420	0.063	0.180
$e^q$	0.420	0.055	0.195
$f^{\perp q}$	0.420	0.063	0.200
$f_4^{q}$	–	0.063	0.235

**Fig. 4:**
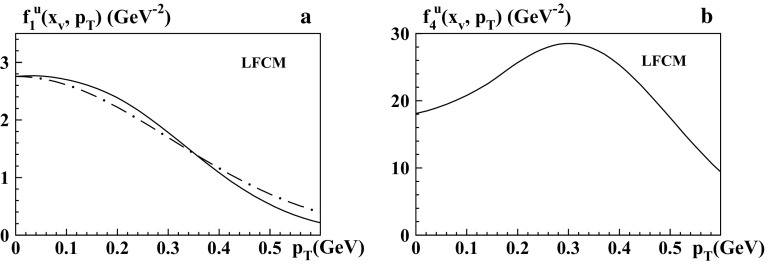
**a** $f_1^u(x_v, p_T)$ at $x_v=0.5$ as functions of $p_T$. The *solid curves* show the predictions from the LFCM, while the *dashed-dotted curves* are the respective Gaussian approximations from Eq. (20) with the Gauss widths in Table 1b. **b** $f_4^u(x_v, p_T)$ at $x_v=0.5$ as functions of $p_T$. We do not show results for $e^q(x,p_T)$ and $f^{\perp q}(x,p_T)$, which differ merely in the overall normalization but exhibit the same $p_T$-dependence as $f_1^q(x,p_T)$

**Fig. 5:**
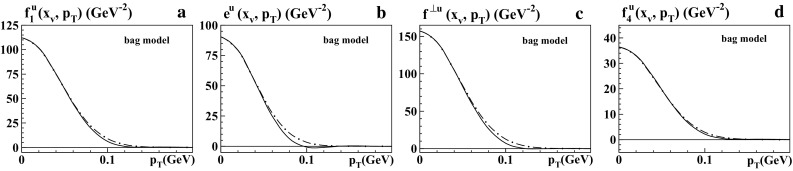
Bag model results for pionTMDs at $x_v=0.5$ as functions of $p_T$ at low scale: **a** $f_1^q(x,p_T)$, **b** $e^q(x,p_T)$, **c** $f^{\perp q}(x,p_T)$, **d** $f_4^q(x,p_T)$. The *solid curves* show the predictions from the bag model, while the *dashed-dotted curves* are the respective Gaussian approximations from Eq. (20) with the Gauss widths in Table 1b

In Table 1a we show the results from the LFCM of the pion for $\langle p_T^{ }\rangle$, $\langle p_T^2\rangle ^{1/2}_{ }$ and the ratio $R_G$. Although $R_G$ is very close to unity for all TMDs, the Gaussian Ansatz is only a rough approximation for $f_1^q$, $e^q$, $f^{\perp q}$ and not applicable at all for $f_4^q$, as shown in the right panel of Fig. 4.

A more reliable test for the applicability of the Gaussian Ansatz can be performed by introducing a different definition of $\langle p_{T,v}^2\rangle$ [55], which is adjusted such that one obtains (if it is possible) a useful approximation of the true $p_T$-dependence of a TMD $j(x,p_T)$ at a given value of (valence-) *x* in terms of the Gaussian Ansatz as

20 $$\begin{aligned} j(x_v,p_T) \approx j(x_v,0) \; \exp \biggl (-\frac{p_T^2}{\langle p_{T,v}^2\rangle }\biggr ). \end{aligned}$$

Although this definition is *x*-dependent, typically the *x*-dependence is weak in the valence-*x* region where quark models are applicable [55]. For definiteness, we choose the value $x_v=0.5$ for the pion as a reference point where $f_1^q(x)$ exhibits a peak in most models.

**Fig. 6:**
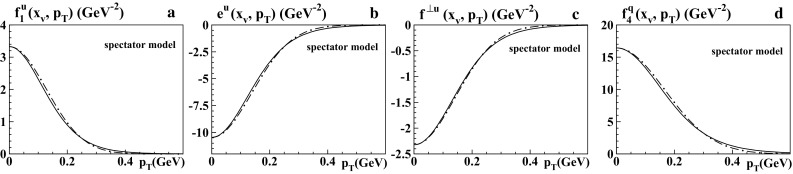
Spectator model results for pion TMDs at $x_v=0.5$ as functions of $p_T$ at low scale: **a** $f_1^q(x,p_T)$, **b** $e^q(x,p_T)$, **c** $f^{\perp q}(x,p_T)$, **d** $f_4^q(x,p_T)$. The *solid curves* show the predictions from the spectator model for $\alpha =3$ model, while the *dashed-dotted curves* are the respective Gaussian approximations from Eq. (20) with the Gauss widths in Table 1b

In Table 1b the second column displays the results from LFCM of the pion for $\langle p_{T,v}^2\rangle ^{1/2}$ of $f_1^q$, $e^q$, $f^{\perp q}$, where the Gaussian approximation is rough but still makes sense; see Fig. 4. These numbers deviate significantly from the results for $\langle p_T^2\rangle ^{1/2}$ in Table 1a. The important lesson is that the “$R_G$-test” is only a necessary but not a sufficient condition for the usefulness of the Gaussian approximation. Using the definition (20), we can also directly compare all models; see the Figs. 5, 6 and other columns in Table 1b. (Notice that the definitions (18) would not be useful in the bag model, where the integrations over *x* in general include unphysical contributions, cf. footnote 2 and the discussion in Sect. 5.2.) Comparing the models we see that the predictions for the widths vary significantly from model to model. Notice that in the LFCM and the spectator model the physical scale is set by the constituent quark mass, and the widths tend to be broader. In contrast to this, in the bag model the widths $\langle p_{T,v}^2\rangle ^{1/2}$ of the pion are substantially smaller. The reason is that the only dimensionful parameter in the bag model (here we work with massless “current quarks” confined in the bag) is the pion mass $m_\pi$, which is rather small.

Finally, for comparison we show in Table 2 the same information as in Table 1b but for the nucleon in which case $x_v=0.3$ is a more appropriate choice as this is where $f_1^q(x)$ peaks in quark models. The nucleon results in Table 2 are from Ref. [25].^4^

The comparison of the results for pion and nucleon in Table 2 is very interesting. We see that the three models make three different predictions. In the LFCM the $p_T$-distributions in the pion are broader than those in the nucleon. In the bag model the situation is opposite. In the spectator model the two hadrons have comparable Gaussian widths. Currently these predictions cannot be tested except for the case of $f_1^q(x,p_T)$, where phenomenological studies indicate that the $p_T$-distribution in $f_1^q(x,p_T)$ of the pion is broader than in the nucleon [57]. This is in qualitative agreement with the predictions of the LFCM in Table 2. One should keep in mind, though, that the phenomenological result was inferred from Drell–Yan data at center-of-mass energies of $\sqrt{s}\sim 23\,\mathrm{GeV}$ and refers to scales $Q > 4\,\mathrm{GeV}$ above the charmonium resonance region [57].

**Table 2:** For comparison, the same as Table 1b but for the nucleon and at $x_v=0.3$. Notice that in the LFCM of the nucleon and the bag model the widths for *u*- and *d*-flavors are the same, but not in the spectator model of the nucleon

Nucleon	LFCM	Bag	Spectator (*u* / *d*)
TMD	$\langle p_{T,v}^2\rangle ^{1/2}$	$\langle p_{T,v}^2\rangle ^{1/2}$	$\langle p_{T,v}^2\rangle ^{1/2}$
$f_1^{q}$	0.240	0.280	0.200/0.270
$e^q$	0.240	0.230	0.160/0.180
$f^{\perp q}$	0.240	0.270	0.180/0.230
$f_4^{q}$	0.350	0.170	0.180/0.250

In contrast to this the LFCM results refer to a low scale $\mu _0 \sim 0.5\,\mathrm{GeV}$. For a more quantitative comparison it is necessary to take carefully evolution effects into account.

## Conclusions

We have studied in constituent model frameworks the ${\mathsf {T}}$-even TMDs of the pion focusing on higher twist, with the goal to establish common features, investigate the origins of tilde terms, and compare the results to the description of unpolarized TMDs in the nucleon. To avoid bias and minimize model dependence, we investigated several constituent models, including the LFCM, bag and spectator models. The results give interesting insights on the internal structure of the pion in the valence-*x* region.

Our focus was on the aspects related to the modeling of 2-body dynamics of the $q\bar{q}$-pair in the pion as opposed to the 3-body dynamics in the nucleon state. The theoretical expressions and numerical results for all higher-twist pion TMDs $e^q$, $f^{\perp q}$, $f_4^q$ from the LFCM and bag model are new, and so are the spectator model expressions and results for $f_4^q$ (in that model expressions for $e^q$, $f^{\perp q}$ were quoted in [51] but numerical results have not been presented previously).

We addressed the question of how genuine QCD interaction-dependent terms contribute to higher-twist TMDs and are modeled in constituent frameworks. In LFCM the hadron states are obtained from a light-front Fock-space expansion in terms of free on-mass-shell parton states, with the essential QCD bound-state information encoded in the LFWF. Each constituent parton state obeys the free equation of motion. Therefore, certain unintegrated relations among TMDs that are valid in free quark models are naturally supported in this approach for both the pion and the nucleon cases, but not all. In particular, relations involving the twist-4 unpolarized TMD $f^q_4$ are not satisfied for the pion, confirming the results obtained in the nucleon case. A fully consistent description of $f_4^q(x)$ in light-front formalism requires the inclusion of zero modes or higher Fock states which go beyond the scope of the LFCM. Due to the academic character of the twist-4 function $f^q_4$ this is of no relevance for practical applications.

For comparison we discussed results for pion TMDs in bag and spectator model. We found that the three models make different predictions especially for higher-twist TMDs. We also explored to which extent the approaches are compatible with a Gaussian shape of the transverse momentum distributions, and found that all model results can be reasonably approximated by a Gaussian $p_T$-shape, except for $f_4^q$ in the LFCM model. In contrast to the bag model and the spectator model, the LFCM predicts broader $p_T$ distributions in the pion than in the nucleon, which is in qualitative agreement with phenomenology. This may indicate that a more realistic description of the pion structure is achieved in the light-front approach than in the other models. More data and phenomenological studies are needed to clarify the situation.

In the quark models discussed in this work, the pion was treated on the same footing as other hadrons, i.e. as a particle composed of the respective constituent degrees of freedom. It has to be regarded as a limitation that these models do not account for the nature of the pion as a Goldstone boson of chiral symmetry breaking. In view of the importance of chiral symmetry breaking, one may wonder to which extent we can trust the picture of the pion structure deduced from such models. We do not know the answer, but recently encouraging observations were made in this regard [25]. In the nucleon chiral symmetry breaking effects were shown to have profound consequences for the sea quark structure, but far less so for valence distributions [58]. In fact, the description of valence-quark distributions in chiral models [58] is qualitatively similar to those obtained in quark models [51, 55]. We are not aware of any argument why this situation should be fundamentally different in the pion case, though it has not yet been investigated and remains an interesting question to address. Another argument in favor of modeling pions and nucleons on the “same footing” in constituent approaches is based on the observation that pion and nucleon have similar sizes. In quark models like LFCM or spectator model, the scale for that is set by the constituent quark mass which also governs the $p_T$-behavior of valence-quark TMDs. As a last encouraging observation, let us mention that in the LFCM a phenomenologically rather successful description of the leading-twist pion structure (including the $\mathsf T$-odd Boer–Mulders function) was obtained [30]. It of course remains to be tested in future studies whether this success continues beyond leading twist.

Our results will provide useful guidelines for the interpretation of Drell–Yan data from pion-nucleon collisions, which are currently under study at the COMPASS experiment at CERN. These data are expected to provide important insights on the (spin) structure of the nucleon. At the same time, these data will provide the unique opportunity to gain valuable insights on the structure of the pion at both leading and subleading twist. In fact, both aspects are tightly connected, and one can view it either way: the pion is used as a tool to investigate the spin structure of the nucleon, and polarized nucleons are used to shed new light on the structure of the pion. In any case, a good understanding of the pion structure is indispensable and worth exploring for its own sake.

## Figures and Tables

**Fig. 7:**
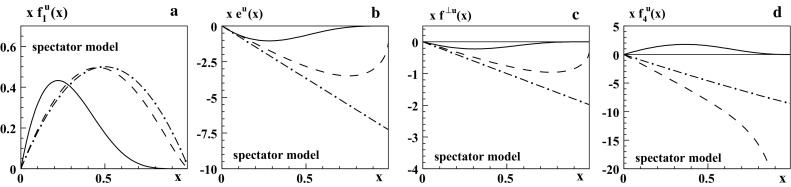
Integrated TMDs of up quarks in the pion from the calculation in the spectator model of the pion [51] with $\alpha =1$ (*dashed-dotted curves*), $\alpha =1.2$ (*dashed curves*) and $\alpha =3$ (*solid curves*)

## Appendix A: Bag model in detail

In this appendix we include the bag model expressions for pion TMDs and PDFs to make this work self-contained, then we discuss the sum rules for the twist-3 function $e^q(x)$ and investigate the symmetries of PDFs.

### A.1 Expressions for TMDs in bag model

The bag model expressions for the pion TMDs,

21 $$\begin{aligned}&f_1^q(x,p_T) = N_q\; A\, \biggl [t_0^2(p) +2\,\frac{p_z}{p}\,t_0(p) t_1(p) +t_1^2(p) \biggr ], \nonumber \\&e^q(x,p_T) = N_q\; A\, \biggl [t_0^2(p) -t_1^2(p) \biggr ], \nonumber \\&f^{\perp q}(x,p_T) = N_q\; A\, \biggl [2\,\frac{M_\mathrm{had}}{p}\,t_0(p) t_1(p) \biggr ], \nonumber \\&f_4^q(x,p_T) = N_q\,\frac{A}{2} \biggl [t_0^2(p) -2\,\frac{p_z}{p}\,t_0(p) t_1(p) +t_1^2(p) \biggr ]\nonumber \\ \end{aligned}$$

with $p=\sqrt{p_z^2+p_T^2}$ and $p_z$ and hadron mass $M_\mathrm{had}$ given by

22 $$\begin{aligned} p_z = \left( x-\frac{3}{4n}\right) M_\mathrm{had},\quad M_\mathrm{had} = \frac{4}{3}\,n\,\frac{\omega }{R_\mathrm{bag}}, \end{aligned}$$

coincide with those for unpolarized nucleon TMDs [25, 55], if one considers the flavor structure, e.g. $$N_u=N_{\bar{d}}=1$$ for $$\pi ^+$$, and writes the normalization constant *A* in a way valid for mesons ($$n=2)$$ and baryons ($$n=3$$) as follows:

23 $$\begin{aligned} A=\frac{M_\mathrm{had}\omega ^3R_0^3}{4\pi ^2(\omega -1)\sin ^2\omega }\;, \end{aligned}$$

where $\omega \approx 2.04$ is the dimensionless “frequency” of the lowest bag eigenmode. In practice one uses the physical hadron mass for $M_\mathrm{had}$ and adjusts the bag radius accordingly. The functions $t_{l}(p)$ in Eq. (21) can be expressed in terms of the spherical Bessel functions $j_l$ with $l=0,1$ as $t_{l}(p)=\int _0^1\mathrm {d}u\,u^2j_l(upR_\text {bag})\,j_l(u\omega )$.

The bag model expression for $f_1^q(x,p_T)$ of the pion was also derived in [59].

### A.2 Sum rules for $e^q(x)$ in bag model

The sum rules (3c), (3d) can be evaluated analytically in the bag model, and one finds for massless quarks

24a $$\begin{aligned} \int \mathrm {d}x\, e^q(x)&=\frac{N_q}{2(\omega -1)}, \end{aligned}$$

24b $$\begin{aligned} \int \mathrm {d}x\,x\,e^q(x)&=\frac{N_q}{2(\omega -1)}\,\frac{3}{4n}. \end{aligned}$$

This is in contrast to QCD, where in the chiral limit the sum rule in Eq. (24a) should diverge and the one in Eq. (24b) should vanish. These results reflect that the MIT bag model is at variance with chiral symmetry [31]. The problem can be traced back to the bag boundary condition, which breaks chiral symmetry. A trivial cure to this problem is to remove the bag boundary.^5^ In this way one restores free quarks which comply with chiral symmetry in the massless limit. However, in this way one also removes the only interaction in this model, and all tilde terms [25]. In particular, in this way $e^q(x,p_T)$ vanishes, as it is a pure bag-boundary effect [31]. Interestingly, this in itself is consistent, because from the general decomposition in Eq. (6a) we see that in the chiral limit and in the absence of interactions one obtains $x\,e^q(x,p_T)=0$, although this does not necessarily imply that $e^q(x,p_T)$ itself must vanish as it could contain a $\delta (x)$-contribution [32, 33]. Notice, however, that Eq. (24a) is within the model consistent, and provides the correct bag contribution to sigma term as can be seen from the cloudy bag model study of the nucleon sigma term in Ref. [61].

It is interesting to confront (24a), (24b) with the sum rules $\int \mathrm {d}x\,f_1^q(x)=N_q$ and $\int \mathrm {d}x\,x\,f_1^q(x)=N_q/n$ showing that the bag model predicts that at low scale $e^q(x)$ is concentrated toward the region of lower *x* as compared to $f_1^q(x)$,

25 $$\begin{aligned} \frac{\int \mathrm {d}x\,x\;e^q(x)}{\int \mathrm {d}x\,e^q(x)} = \frac{3}{4}\;\frac{1}{n}\;\;\; \mathrm{vs.}\;\; \frac{\int \mathrm {d}x\,x\;f_1^q(x)}{\int \mathrm {d}x\,f_1^q(x)} = \frac{1}{n}. \end{aligned}$$

### A.3 Symmetries of PDFs in bag model

From Eq. (21) one obtains the following expressions for the PDFs (where $p_z$ retains its meaning as defined in Eq. (22), but *p* denotes a dummy integration variable in this section):

26 $$\begin{aligned} f^q_1(x)= & {} N_q\,2\pi \,A\int _{p_z}^\infty \mathrm {d}p\;p\nonumber \\&\times \biggl [t^2_0(p)+2\,\frac{p_z}{p}\,t_0(p)t_1(p)+t_1^2(p)\biggr ],\nonumber \\ e^q(x)= & {} N_q\,2\pi \,A\int _{p_z}^\infty \mathrm {d}p\;p \biggl [t^2_0(p)-t_1^2(p)\biggr ],\nonumber \\ f^{\perp q}(x)= & {} N_q\,2\pi \,A\int _{p_z}^\infty \mathrm {d}p\;p \biggl [2\,\frac{M_\mathrm{had}}{p}\,t_0(p)t_1(p)\biggr ],\nonumber \\ f^q_4(x)= & {} N_q\,2\pi \,A\int _{p_z}^\infty \mathrm {d}p\;p \nonumber \\&\times \biggl [t^2_0(p)-2\,\frac{p_z}{p}\,t_0(p)t_1(p)+t_1^2(p)\biggr ]. \end{aligned}$$

To understand the exact and approximate symmetries of the PDFs in the bag model, we need to recall that $t_0(p)$ is an even function of *p*, while $t_1(p)$ is an odd function of *p*. This implies that the integrands of all PDFs are odd functions of *p*, i.e. in all cases the identity $\int _{-\kappa }^{\kappa } \dots = 0$ holds where the dots indicate the respective integrands. If we choose $\kappa = p_z$ we immediately conclude that for all PDFs one can equally well replace the lower integration limit by $(-p_z)$ or simply by $|p_z|$. This will be useful in the following.

The exact properties of $e^q(x)$ can be derived as follows. We can find the maximum of $e^q(x)$ by differentiating

27 $$\begin{aligned} \frac{\mathrm {d}}{\mathrm {d}x}\,e^q(x)= & {} -N_q\,2\pi \,A\,M_\mathrm{had}\;p_z\left[ t^2_0(p_z)-t_1^2(p_z)\right] \mathop {=}\limits ^{!} 0 \nonumber \\\Leftrightarrow & {} \mathrm{(i)}\;\; p_z\mathop {=}\limits ^{!} 0 \;\; \mathrm{or} \; \; \mathrm{(ii)}\;\;t^2_0(p_z)\mathop {=}\limits ^{!}t_1^2(p_z). \end{aligned}$$

The condition (i) yields the position of the global maximum (as one can confirm by inspecting the second derivative)

28 $$\begin{aligned} x_\mathrm{max}=\frac{3}{4n}\,. \end{aligned}$$

For completeness we remark that condition (ii) leads to many more extrema with most of them appearing in unphysical regions of *x*.

Next, as we have seen above, the lower integration limit in Eq. (26) can also be chosen as $|p_z|$. Since no factor of $p_z$ appears in its integrand, this means that $e^q(x)$ is a function symmetric under $p_z\mapsto -p_z$, i.e. it satisfies the exact symmetry

29 $$\begin{aligned} e^q(2x_\mathrm{max}-x) = e^q(x),\quad x_\mathrm{max}=\frac{3}{4n}. \end{aligned}$$

The above derivation can be repeated step by step with $f^{\perp q}(x)$. Although it has a different shape, this PDF exhibits a global maximum at the same position as $e^q(x)$ and satisfies also the same exact symmetry

30 $$\begin{aligned} f^{\perp q}(2x_\mathrm{max}-x) = f^{\perp q}(x),\quad x_\mathrm{max}=\frac{3}{4n}. \end{aligned}$$

For $f_1^q(x)$ and $f_4^q(x)$ the situation is different, and no exact symmetry of the above kind exists due to the appearance of the explicit factor of $p_z$ in their integrands in Eq. (26). What one can derive in this case is the exact relation

31 $$\begin{aligned} f^q_1(x)=2f^q_4\biggl (\frac{3}{2n}-x\biggr ), \end{aligned}$$

though the value $x=\frac{3}{2n}$ is not related to the maxima of $f^q_1(x)$ or $2f^q_4(x)$. One interesting application of Eq. (31) is that it immediately follows that $\int \mathrm {d}x\,f_1^q(x)=\int \mathrm {d}x\,2f_4^q(x)$ if one recalls the remarks in footnote 2.

One can use the above method to find the maximum of $f_1^q(x)$. The unpolarized function has its maximum at that value of *x* which solves the integral equation

32 $$\begin{aligned} p_z \left( t_0(p_z)+t_1(p_z)\right) ^2 = 2 \int _{p_z}^\infty \mathrm {d}p\;t_0(p)\,t_1(p), \end{aligned}$$

where *x* appears implicitly in $p_z$; see Eq. (22). The solution can be found numerically and reads

33 $$\begin{aligned} x_\mathrm{max} = (1.00534\dots )\times \frac{1}{n}. \end{aligned}$$

This is numerically very close to $x\approx \frac{1}{n}$ and an intuitive result, see Sect. 5.2. There is also an approximate symmetry

34 $$\begin{aligned} f_1^q(\frac{2}{n}-x) \approx f^q(x), \end{aligned}$$

which for $n=2$ is satisfied to within $\mathcal{O}(1\,\%)$ accuracy for $x\in [0,1]$ (the approximate symmetry $f_1^q(2x_\mathrm{max}-x) \approx f_1^q(x)$ is much better in the vicinity of $x_\mathrm{max}$ but interestingly overall somewhat worse).

The situation for $f_4^q(x)$ is similar to that of $f_1^q(x)$ except for the difference that the maximum appears at $x_\mathrm{max}\approx \frac{1}{2n}$. More precisely, for $f_4^q(x)$ one deals with the integral relation

35 $$\begin{aligned} p_z \left( t_0(p_z)-t_1(p_z)\right) ^2 = -\,2 \int _{p_z}^\infty \mathrm {d}p\;t_0(p)\,t_1(p)\, , \end{aligned}$$

and the solution is

36 $$\begin{aligned} x_\mathrm{max} = (0.989327\dots )\times \frac{1}{2n}\,. \end{aligned}$$

## Appendix B: Spectator model in detail

In this appendix we review results for $f_1^q(x,p_T)$, $e^q(x,p_T)$ and $f^{\perp q}(x,p_T)$ from [51], derive in addition the expression for $f_4^q(x,p_T)$, and discuss the momentum sum rule, and parameter fixing in the spectator model.

### B.1 Expressions for TMDs in spectator model

In the quark–spectator–antiquark model of the pion, the correlator (2) is evaluated as follows:

37 $$\begin{aligned}&\int \frac{\mathrm {d}z^-\mathrm {d}^2z_T}{2(2\pi )^3} \, e^{i p \cdot z} \, \langle P|\overline{\psi }(0)\Gamma \psi (z)|P\rangle |_{z^+=0} \nonumber \\&\quad \equiv \left. \frac{\mathrm {Tr}[{\tilde{\Phi }}^q\Gamma ]}{4(1-x)P^+} \right| _{p^2=xm^2_\pi -\tfrac{p^2_T+xM^2_R}{1-x}}, \end{aligned}$$

where

38

with $g(p^2)$ a form factor. This form factor is often assumed to be [62]

39 $$\begin{aligned} g(p^2)=N\,\frac{p^2-m^2_q}{|p^2-\Lambda ^2|^\alpha }, \end{aligned}$$

where $\Lambda$ is a cut-off parameter and *N* is a normalization constant. This choice has the advantage of killing the pole of the quark propagator.

The results for the quark TMDs of the pion read

40 $$\begin{aligned} f_1^{q}(x,p_T)= & {} B\,\frac{(m_q+xm_\pi )^2+p^2_T}{1-x},\nonumber \\ e^{q}(x,p_T)= & {} \frac{B}{(1-x)^2}\, \left[ (1-x)(m_q+xm_\pi )(m_q+m_\pi )\right. \nonumber \\&\left. -M^2_R(x+\tfrac{m_q}{m_\pi }) -(1+\tfrac{m_q}{m_\pi })p^2_T\right] , \nonumber \\ f^{\perp q}(x,p_T)= & {} \frac{B}{(1-x)^2}\, \left[ (1-x^2)m^2_\pi +2m_qm_\pi (1-x)\right. \nonumber \\&\left. -M^2_R-p^2_T\right] ,\nonumber \\ f_4^{q}(x,p_T)= & {} \frac{B}{2(1-x)^2}\, \left\{ (1-x)\left[ (m_q+m_\pi )^2-M^2_R\right] \right. \nonumber \\&\left. +\frac{p^2_T+M^2_R}{m^2_\pi } \left[ \frac{p^2_T+M^2_R}{(1-x)}-2m_\pi m_q-(1+x)m^2_\pi \right] \right\} ,\nonumber \\ \end{aligned}$$

where we introduced for convenience

41 $$\begin{aligned} B=\frac{N^2}{2(2\pi )^3} \left[ \frac{1-x}{p^2_T+\lambda ^2_R(x)}\right] ^{2\alpha } \end{aligned}$$

with

42 $$\begin{aligned} \lambda ^2_R(x)=(1-x)\Lambda ^2+xM^2_R-x(1-x)m^2_\pi . \end{aligned}$$

The results for the integrated TMDs read

43 $$\begin{aligned} f_1^{q}(x)= & {} B'\left[ 2(\alpha -1)(m_q+xm_\pi )^2+\lambda ^2_R(x)\right] , \nonumber \\ e^{q}(x)= & {} \frac{B'}{1-x} \biggl \{2(\alpha -1)(x+\tfrac{m_q}{m_\pi }) [(1-x)(m_q+m_\pi ) m_\pi \nonumber \\&-M^2_R] -(1+\tfrac{m_q}{m_\pi })\lambda ^2_R(x)\biggr \},\nonumber \\ f^{\perp q}(x)= & {} \frac{B'}{1-x}\biggl \{2(\alpha -1)[ (1-x^2)m_\pi ^2+2m_qm_\pi (1-x)\nonumber \\&-M_R^2] -\lambda ^2_R(x)\biggr \},\nonumber \\ f_4^{q}(x)= & {} \frac{B'}{2(2\alpha -3)m_\pi ^2(1-x)^2}\, \biggl \{\lambda ^4_R(x)- (2\alpha -3)\nonumber \\&\times \lambda ^2_R(x) \left[ 2m_qm_\pi (1-x)+(1-x^2)m_\pi ^2-2M_R^2\right] \,\nonumber \\&+ 2(\alpha -1)(2\alpha -3)\left[ m_\pi ^2(1-x)^2[(m_q+m_\pi )^2-M_R^2]\right. \nonumber \\&\left. -M_R^2\left[ 2m_qm_\pi (1-x)+(1-x^2)m_\pi ^2-M_R^2\right] \right] \biggr \}, \end{aligned}$$

where we introduced

44 $$\begin{aligned} B'=\frac{N^2}{8(2\pi )^2(2\alpha -1)(\alpha -1)} \left[ \frac{1-x}{\lambda ^2_R(x)}\right] ^{2\alpha -1}. \end{aligned}$$

### B.2 Limit $\alpha \rightarrow 1$ and momentum sum rule

In the limit of $\alpha \rightarrow 1$ and $M_R\rightarrow m_q$ the PDFs reduce to, see Ref. [51],

45a $$\begin{aligned}&f_1^q(x) \mathop {=}\limits ^{\alpha \rightarrow 1} 2(1-x), \end{aligned}$$

45b $$\begin{aligned}&e^q(x) \mathop {=}\limits ^{\alpha \rightarrow 1} -2 \left( 1 +\frac{m_q}{m_\pi }\right) ,\end{aligned}$$

45c $$\begin{aligned}&f^{\perp q}(x) \mathop {=}\limits ^{\alpha \rightarrow 1} -2,\end{aligned}$$

45d $$\begin{aligned}&f^q_4(x) \mathop {=}\limits ^{\alpha \rightarrow 1} \frac{2m_q^2-2m_qm_\pi (1-x)-m_\pi ^2(1-x^2) -2\lambda ^2_R(x)}{m^2_\pi (1-x)}. \end{aligned}$$

The result (45a) is interesting, as it implies that $x\,f_1^q(x)$ is symmetric under the exchange $x \leftrightarrow (1 - x)$. But except for (45a) the results are unphysical, since the distributions do not vanish for $x\rightarrow 1$. Choices of $\alpha$ leading to acceptable results for all TMDs are discussed in Appendix B.3.

Although the limit $\alpha \rightarrow 1$ in (45) is not acceptable for all TMDs, the results  (45a) is useful for illustrative purposes. We shall work with this result to discuss the sum rules (3a) and (3b). The valence sum rule (3a) is satisfied (in the limit $\alpha \rightarrow 1$ and for $\alpha \ne 1$) though this is by construction, as the normalization constant *N* is chosen adequately. But the momentum sum rule (3b) is not valid. In the limit given by Eq. (45a) we obtain $\sum _q\int \mathrm {d}x\,x\,f_1^q(x)=\frac{2}{3}$, where the sum goes over, e.g., the constituents $q=u,\,\bar{d}$ of the positive pion.

Taken literally this result means the constituents carry only $\frac{2}{3}$ of the hadron momentum. The deeper reason for this paradox can be traced back to the fact that the spectator model is an incomplete system as it does not account for the forces that would bind the constituents to form a proper hadronic bound state which is essential^6^ to comply with the momentum sum rule [64]. We note that $\sum _q\int \mathrm {d}x\, x\,f_1^q(x)<\frac{2}{3}$ for $\alpha >1$. Notice that in semi-phenomenological models based on the rainbow-ladder truncation of the QCD Dyson–Schwinger equations, one finds that valence quarks carry $\frac{2}{3}$ of the pion momentum [66, 67]. One could therefore be tempted to argue that the spectator model describes TMDs at somewhat higher scales, where valence quarks do not carry anymore $100\,\%$ of the hadron momentum. However, this is phenomenologically not supported [51] and must not distract from the fact that this model lacks the dynamics to form a consistent bound state.

### B.3 Fixing of model parameters

In the spectator model it is a priori not clear which value of $\alpha$ should be chosen in the form factors in Eq. (39). In Fig. 7 we therefore show the results from the spectator model of the pion for $xf_1^q(x)$, $xe^q(x)$, $xf^{\perp q}(x)$, and $xf_4^q(x)$ for different values of $\alpha$. We fix $M_R=m_q$, with $m_q=360$ MeV.

The dashed-dotted lines in Fig. 7 show results for $\alpha =1$ chosen in Ref. [51] for $f_1^q(x)$. This is not acceptable for the other TMDs which with this choice do not vanish for $x\rightarrow 1$; see Appendix B.2. For $\alpha \ne 1$ the TMDs depend also on the cut-off $\Lambda$, that is taken equal to 0.4 GeV as in Ref. [51]. For $\alpha > 1$ one obtains $e^q(x)$ and $f^{\perp q}(x)$, which vanish as $x\rightarrow 1$. To illustrate this point, we plot the results (dashed curves) for $\alpha =1.2$ in Fig. 7. However, with this choice $f_4^q(x)$ is negative, violates the inequality (4b), and even diverges as $x\rightarrow 1$. Both artifacts can be fixed by choosing $\alpha > \frac{3}{2}$. The smallest integer value $\alpha = 2$ would give a very large result for $f_4^q(x)$ with $\int \mathrm {d}x\,f_4^q(x) = 10.3$ strongly exceeding the sum rule (3e). We plot therefore in Fig. 7 the results for $\alpha = 3$ (as solid lines) where $\int \mathrm {d}x\,f_4^q(x) = 3.6$ overestimates (3e) less drastically (recall that the sum rule (3e) cannot be satisfied for any $\alpha$).

We remark that one could vary the model parameters much more than that, e.g. one could vary the cut-off or relax the assumption that the spectator mass should be associated with the constituent quark mass $m_q$. But in this work we shall content ourselves with the insight on the model dependence from the variation with respect to $\alpha$ in Fig. 7. The results shown in the main text were obtained for $\alpha =3$.

**Table 3:** Off-shellness effects in the spectator model of pion (a) and nucleon (b) at selected values of *x* and $p_T$. If the active parton was onshell, the ratio $p^2/m_q^2$ would be unity and the tilde functions in Eqs. (6a)–(6c) would be absent

(a) Pion	$p^2/m_q^2$		
$p_T$ (GeV)	0.1	0.2	0.3
*x*			
0.1	−0.18	−0.44	−0.87
0.2	−0.32	−0.61	−1.09
0.3	−0.49	−0.82	−1.38

(b) Nucleon	$p^2/m_q^2$		
$p_T$ (GeV)	0.1	0.2	0.3
*x*			
0.1	0.05	−0.21	−0.64
0.2	0.03	−0.26	−0.74
0.3	−0.18	−0.51	−1.06

### B.4 Off-shellness effects and tilde terms

The explicit expression for the tilde terms in the spectator model with $M_R=m_q$ read

$$\begin{aligned} x \tilde{e}^q(x,p_T)= & {} B\,\frac{p^2-m_q^2}{1-x}\,\left( x+\frac{m_q}{m_\pi }\right) ,\\ x \tilde{f}^{\perp q}(x,p_T)= & {} B\,\frac{p^2-m_q^2}{1-x},\\ x^2 \tilde{f}^q_4(x,p_T)= & {} B\,\frac{p^2-m_q^2}{1-x}\,\frac{1}{2m^2_\pi } \biggl [[(m_q+xm_\pi )^2+p^2_T]\\&+x(1-x)m^2_\pi -\frac{x(p^2_T+m^2_q)}{1-x}\biggr ]. \end{aligned}$$

These terms arise from the off-shellness effects $p^2\ne m_q^2$. We recall that in the spectator model the virtuality of the parton is given by $p^2 = xM^2_\mathrm{had}-(p^2_T+xM^2_R)/(1-x)$. The results for the off-shellness effects at different values of *x* and $p_T$ are shown in Table 3a, b for the case of pion and nucleon, respectively. (The nucleon results are obtained with the axial diquark mass, with the parameters used in [25, 51].) We observe that these off-shellness effects are larger for the pion than for the nucleon, and the difference is more pronounced for small *x* and moderate $p_T$.

In particular, the tilde terms in $e^q(x)$ and $f^{\perp q}(x)$ are not only sizable but also negative, and in fact overwhelm the contributions of the positive $f_1^q(x)$ in Eqs. (6a) and (6b). This explains why $e^q(x)$ and $f^{\perp q}(x)$ are negative in the spectator model of the pion—in contrast to the other models. This feature is qualitatively different from the nucleon case, where the tilde terms could be viewed as “corrections” albeit not necessarily small ones [25]. The reason for that is that in the pion case the constituent quark mass is larger than the hadron mass, i.e. off-shellness effects are automatically more extreme than in the nucleon case. As a result, the spectator model of the pion does not support the Wandzura–Wilczek type approximation [68] consisting in a neglect of tilde terms.

## Footnotes

1 We stress that such relations are valid only in quark models (or for the (academic) TMDs with straight gauge links implemented in lattice calculations [35]), and should be distinguished from the LIRs in collinear twist-3 formalism recently derived in Ref. [36].

2 For that it is crucial to extend the integrals over the whole *x*-axis including negative *x* and the regions $|x|>1$. At negative *x* the TMDs describe sea quarks where the bag results violate positivity (4a), (4b) in the nucleon case. The non-zero (albeit numerically small) support in the regions $|x|>1$ is a technical problem in models where corrections due to center-of-mass motion have to be applied. These issues are known in the bag model of the nucleon and not specific to the pion.

3 In the model calculation of Ref. [30] the mass of the constituent quark in the nucleon was chosen as $m_q=0.263$ GeV.

4 We would like to use this occasion to correct a numerical mistake in the second column of Table 2 in Ref. [25], where the widths in the LFCM of the nucleon were incorrectly scaled by a factor of $1/\sqrt{\pi }$. The second column of Table 2 in this work gives the correctly scaled values. This correction does not affect any of the conclusions of Ref. [25].
